# Viral co-infections in hospitalised COVID-19 patients recruited to the ISARIC WHO CCP-UK study

**DOI:** 10.1093/ofid/ofac531

**Published:** 2022-10-10

**Authors:** Elen Vink, Chris Davis, Alasdair MacLean, David Pascall, Sarah E McDonald, Rory Gunson, Hayley E Hardwick, Wilna Oosthuyzen, Peter JM Openshaw, J Kenneth Baillie, Malcolm G Semple, Antonia Ho

**Affiliations:** Medical Research Council-University of Glasgow Centre for Virus Research, University of Glasgow, Glasgow, UK; Medical Research Council-University of Glasgow Centre for Virus Research, University of Glasgow, Glasgow, UK; West of Scotland Specialist Virology Centre, NHS Greater Glasgow and Clyde, Glasgow, UK; MRC Biostatistics Unit, University of Cambridge, Cambridge, UK; Joint Universities Pandemic and Epidemiological Research (JUNIPER) consortium; Medical Research Council-University of Glasgow Centre for Virus Research, University of Glasgow, Glasgow, UK; West of Scotland Specialist Virology Centre, NHS Greater Glasgow and Clyde, Glasgow, UK; NIHR Health Protection Research Unit in Emerging and Zoonotic Infections, Institute of Infection, Veterinary and Ecological Sciences, Faculty of Health and Life Sciences, University of Liverpool, Liverpool, UK; Roslin Institute, University of Edinburgh, Easter Bush, Edinburgh, UK; National Heart and Lung Institute, Imperial College London, London, UK; Roslin Institute, University of Edinburgh, Easter Bush, Edinburgh, UK; NIHR Health Protection Research Unit in Emerging and Zoonotic Infections, Institute of Infection, Veterinary and Ecological Sciences, Faculty of Health and Life Sciences, University of Liverpool, Liverpool, UK; The Pandemic Institute, University of Liverpool, Liverpool, UK; Medical Research Council-University of Glasgow Centre for Virus Research, University of Glasgow, Glasgow, UK

**Keywords:** SARS-CoV-2, disease severity, respiratory virus, rhinovirus, surveillance, PCR

## Abstract

**Objective:**

To assess the prevalence of viral co-infection in a well-characterised cohort of hospitalised COVID-19 patients, and to investigate the impact of co-infection on disease severity.

**Methods:**

Multiplex real-time polymerase chain reaction testing for endemic respiratory viruses was performed on upper respiratory tract samples from 1002 COVID-19 patients, aged <1 year to 102 years old, recruited to the International Severe Acute Respiratory and Emerging Infections Consortium WHO Clinical Characterisation Protocol UK study. Comprehensive demographic, clinical, and outcome data were collected prospectively up to 28 days post discharge.

**Results:**

A co-infecting virus was detected in 20 (2.0%) participants. Multivariable analysis revealed no significant risk factors for co-infection, though this may be due to rarity of co-infection. Likewise, ordinal logistic regression analysis did not demonstrate a significant association between co-infection and increased disease severity.

**Conclusion:**

Viral co-infection was rare among hospitalised COVID-19 patients in the UK during the first eighteen months of the pandemic. With unbiased prospective sampling, we found no evidence of an association between viral co-infection and disease severity. Public health interventions disrupted normal seasonal transmission of respiratory viruses; relaxation of these measures mean it will be important to monitor the prevalence and impact of respiratory viral co-infections going forward.

### Keywords

SARS-CoV-2, disease severity, respiratory virus, rhinovirus, surveillance, PCR

### Background

SARS-CoV-2 emerged as a new viral pathogen in China at the end of 2019 and has since become pandemic with over 173 million confirmed cases of the associated disease, COVID-19, and nearly 6.4 million deaths worldwide (up to 14th July 2022) [1]. Evidence from existing studies of patients with influenza, respiratory syncytial virus (RSV), and rhinovirus infections suggest that viral co-infections are associated with increased disease severity [2–6]. However, data on the frequency of co-infection in COVID-19 patients and how co-infections impact on disease severity are scarce and variable. Few studies have systematically and prospectively tested for a broad range of respiratory viruses in a patient cohort with comprehensive clinical metadata to allow for analysis of the impact of viral co-infection on disease severity and clinical outcomes.

COVID-19 public health measures such as physical distancing, lockdowns and travel restrictions have resulted in a dramatic reduction in the circulation of other respiratory viruses over the past 2 years [7,8], However, as countries across the world relax restrictions, community transmission of these viruses will likely rise and consequently as will the risk of viral co-infections in COVID-19 patients. Understanding the nature, frequency and impact of co-infections are key to optimise the clinical management of patients, inform vaccination strategies and best practice in infection prevention and control, in addition to guiding healthcare service planning and funding.

In this prospective study, we assessed the prevalence of viral co-infection in a well-characterised cohort of hospitalised COVID-19 patients in the UK by systematically testing upper respiratory tract samples for a broad range of respiratory viruses, and investigated the impact of co-infection on disease severity and clinical outcomes using comprehensive clinical metadata.

## Methods

### Study Setting and Approvals

The International Severe Acute Respiratory and Emerging Infections Consortium (ISARIC) WHO Clinical Characterisation Protocol UK (CCP-UK) study is a prospective cohort study which recruited inpatients from hospitals across the UK (National Institute for Health Research [NIHR] Clinical Research Network Central Portfolio Management System ID 14152) and was conducted by the ISARIC Coronavirus Clinical Characterisation Consortium (ISARIC4C). Ethics approval was given by the South Central-Oxford C Research Ethics Committee in England (13/SC/0149), the Scotland A Research Ethics Committee (20/SS/0028), and the WHO Ethics Review Committee (RPC571 and RPC572; April 2013). The study protocol and further details are available online [9].

### Study Participants

Recruitment of COVID-19 cases to the ISARIC WHO CCP-UK study took place between 5th February 2020 and 28th February 2022 and has previously been described in detail [9]. In brief, demographic, clinical, laboratory, therapeutic, and outcome data were collected for participants with laboratory-confirmed or highly suspected COVID-19 on a standardised case report form. Data were uploaded to a Research Electronic Data Capture Database (REDCap; Vanderbilt University, Nashville, TN USA; hosted by University of Oxford, Oxford, UK) [10]. A subset of ~2500 participants gave consent for the collection of biological samples, including upper respiratory tract (URT) swabs, on days 1, 3, 9 and 28 post-enrolment. URT samples from all time points were not available for every participant due to the need to prioritise clinical care. We included day 1 (or if this was not available, day 3) URT samples only, and excluded participants if their day 1 or day 3 samples were collected more than 14 days after admission or if the participant had nosocomial COVID-19, defined as symptom onset more than 7 days after admission (Figure 1). Participants were also excluded who were SARS-CoV-2 PCR negative.

### Collection and RNA extraction of URT swabs

Samples were collected from study participants using throat or nasal swabs that were placed into viral transport medium and stored at -80°C. URT samples were inactivated and lysed with NucliSENS® Lysis Buffer. Equine arteritis virus (EAV) was used as an internal control (IC) which was introduced into the lysis buffer at this stage. RNA extraction was performed using the fully automated Biomérieux EMAG® platform with negative controls included on each extraction run. Eluates were stored at -80°C prior to testing.

### Multiplex Respiratory PCR Panel

A validated in-house multiplex real-time polymerase chain reaction (PCR) panel for respiratory viruses, developed at the West of Scotland Specialist Virology Centre [11], was used to test 6 μl of extracted RNA for adenovirus, rhinovirus, respiratory syncytial viruses (RSV) A and B, influenza A and B viruses, parainfluenza viruses 1-4, human coronaviruses (hCoV) NL63, 229E and OC43, and human metapneumovirus. Positive controls for all viral targets, and negative controls were included in all PCR runs. RT-PCR was performed on an Applied Biosystems 7500 Fast Real-Time PCR System. The following thermal profile was used: a single cycle of reverse transcription for 10 min at 50°C, 2 min at 95°C for reverse transcriptase inactivation and DNA polymerase activation followed by 40 amplification cycles of 8 seconds at 95°C and 30 seconds at 60°C each (annealing-extension step). Data acquisition occurred at the annealing step of each cycle, and the threshold cycle (Ct) for each sample was calculated by determining the point at which the fluorescence exceeded the threshold limit. A Ct of <40 was considered positive.

### COVID-19 Disease Severity Score

Participants were assigned a disease severity score according to maximum level of respiratory support required and clinical outcome. Categories were based on the World Health Organization (WHO) COVID-19 ordinal scale [12] but restricted to hospitalised patients: 1) no oxygen requirement (WHO score 3); 2) patient requiring oxygen by face mask or nasal prongs (WHO score 4); 3) patient requiring high-flow nasal oxygen (HFNO) or non-invasive ventilation (NIV) (WHO score 5); 4) patients requiring mechanical ventilation (WHO score 6/7) and 5) patients who died within 28 days of admission (WHO score 8).

### Statistical Analysis

Categorical variables are reported as frequencies and percentages. Continuous variables are reported as means and standard deviations or medians and interquartile range (IQRs) based on normality. We investigated the association of demographic factors (age, sex, ethnicity, number of comorbidities, and immune status), clinical characteristics at admission (symptoms, observations, laboratory results, and radiology results), disease severity score, and clinical outcomes (admission to critical care, death with 28 days, and length of stay for survivors), with co-infection status using Fisher's exact test or Kruskal Wallis test, for categorical and continuous non-parametric variables respectively. $p \leq 0.05$ was considered statistically significant. Immunocompromise was classified as clinician defined or those receiving immunosuppressive therapy prior to hospital admission. Comorbidities were defined as those listed in the 4C Mortality Score [13].

Multivariable logistic regression analyses were used to seek associations between co-infection status and critical care admission or death, adjusting for the following variables (chosen *a priori*): age, sex, number of comorbidities, immune status, and timepoint within the study. Timepoint was defined as difference in days between the date of sample collection and the date of the earliest sample in the study, i.e. 5th February 2020. Ordinal logistic regression analysis was used to investigate any association between co-infection status and disease severity score. All analyses were performed using R version 4.1.1 using the *Tidyverse*, *finalfit*, and *MASS* packages with model diagnostics performed using *PResiduals* and *sure* packages.

## Results

This analysis included URT samples from 1002 participants admitted to 36 UK hospitals between 5th February 2020 and 11th June 2021 (Figure 1). The median participant age was 60 years (IQR 49–72) and 622 (62.1%) were male (Table 1). 22 (2.2%) participants were under 18 years of age. 596 (59.4%) participants had one or more comorbidity (Table 1); the commonest comorbidities being diabetes (24.2%) and chronic cardiac disease (19.3%). 169 participants (16.9%) were immunocompromised either due to underlying disease or immunosuppressant medication (Table 1).

Median duration of symptoms at admission was 7 days (IQR 3-10) (Supplementary table 1); most frequently reported symptoms on admission were cough (79.2%), fever (76.0%), and shortness of breath (74.0%), and 60.4% (247/409) of admission chest x-rays revealing pulmonary infiltrates (Table 1). Median duration from symptom onset to sample collection was 9 days (IQR 6-13). 387 (38.7%) participants were admitted to critical care with 263 (26.3%) participants requiring mechanical ventilation. Overall, 171 participants (16.9%) died and the median length of hospital admission for those discharged alive was 8 days (IQR 5-16) (Table 1). All severity score groups were well represented in the patient cohort (Table 1).

A respiratory virus other than SARS-CoV-2 was detected in 20 (2.0%) COVID-19 patients. Rhinovirus and parainfluenza virus 4 were each identified in 4 (0.4%), hCoV NL63 and parainfluenza virus 2 in 3 participants (0.3%), hCoV OC43 in 2 participants, and Influenza B virus, parainfluenza virus, human metapneumovirus, and RSV were detected in 1 participant each (Figure 2a). No cases of co-infection with more than one additional virus were detected. Participants with viral co-infection were all admitted prior to mid-May 2020 (Figure 2b). All cases of rhinovirus co-infection were detected during March 2020, and the diversity of co-infecting viruses diminished with time (Figure 2c).

The highest proportion of co-infected participants was found in individuals under 18 years, with the lowest proportion in those aged 35 – 50 years, though these differences were not significant (Figure 3). Age (median 61.5 years (co-infected) vs. 60.0 years (mono-infected), p=0.89), sex (60.0% vs 62.2% male, p=0.82), number of comorbidities (median 0.5 vs 1.0, p=0.76), or immune status (25.0% vs 16.7% immunocompromised, p=0.36) among COVID-19 patients did not differ by co-infection status (Table 1). Univariable and multivariable analysis demonstrated that the timing of SARS-CoV-2 infection within the pandemic was significantly associated with the risk of co-infection (adjusted odds ratio (aOR) 0.97, 95% CI 0.95-0.99, p = 0.03) (Figure 3) as would be expected given that all cases of co-infection were detected prior to mid-May 2020. No other significant risk factors for co-infection were identified (Figure 3).

Presenting symptoms and laboratory test results on admission did not differ significantly between mono-infected and co-infected participants. However, participants with viral coinfection reported wheeze more often (18.8% vs 7.8%, p=0.13) (Supplementary table 1), and had a higher median white cell count (9.8 vs 7.1 $x10^9$cells/L, p=0.13); driven by a higher neutrophil count (8.0 vs 5.3 $x10^9$cells/L, p = 0.12) (Table 1). There was no observed difference in disease severity (median severity score 2.5 vs 3.0 p=0.75), critical care admission (45.0% vs 38.6%, p=0.64), death (21.0% vs 17.6%, p=0.76), or the length of hospital admission for those discharged alive (8 vs 10 days, p=0.69), between the two groups (Table 1).

Male sex was associated with admission to critical care (aOR 1.81, 95% CI 1.36-2.40, p<0.001) (Figure 4a) whilst age greater than 70 years showed an inverse association (aOR 0.47, 95%CI 0.27–0.83, p=0.009). No association was seen between co-infection status and critical care admission (aOR 1.21, 95% CI 0.47-3.07, p=0.69).

Male sex, age >50 years, and increasing number of comorbidities were all associated with increased mortality in univariate and multivariate analysis (Figure 4b). However, viral coinfection had no impact on mortality (aOR 1.17, 95% CI 0.31-3.65, p=0.80).

Ordinal logistic regression analysis demonstrated a significant association between male sex (OR 1.80, 95% CI 1.41 – 2.30, p<0.001), increasing age (OR 1.01, 95% CI 1.00-1.02, p=0.02), and the presence of one comorbidity (OR 1.43, 95% CI 1.15-1.79, p=0.001) with increasing disease severity score (Table 2, supplementary table 2 & supplementary figure 1), but viral coinfection did not show this association (OR 1.14, 95% CI 0.61–2.14, p=0.68).

## Discussion

Viral co-infections were rare (2%) in our multicentre cohort of hospitalised COVID-19 patients recruited during the first 18 months of the pandemic in the UK, with co-infection associated with a variety of respiratory viruses. This is the first study of this size to systematically test hospitalised COVID-19 patients for a broad range of respiratory viruses and as such provides a more accurate picture of viral co-infections among inpatients during this period.

All co-infections were detected in participants admitted before mid-May 2020, with the majority detected in March 2020, and the diversity of viruses detected falling through April and May 2020. This coincides with the introduction of strict public health measures in the UK including a national lockdown that came into force on 23rd March 2020, and correlates with the dramatic decline in the detection of these viruses by national surveillance programmes between mid-March and mid-April

2020 [14]. Rhinovirus was the most frequently identified co-infecting virus in our cohort and was also the most ubiquitous virus in the UK in February and early March 2020 [14].

The reported prevalence of viral co-infection in published studies [15–40] vary greatly from 0.5% [17,32] to 57.3% [22], with our finding of 2% at the lower end of this range. The majority of existing studies identified a prevalence of less than 8%. Studies that reported higher prevalence did not test participants systematically [16,22,25,27,33,35,40], tested solely for influenza [16,22], used serological methods [22], restricted testing to participants that died [25], or had small study numbers (<500) [22,25,27,33,35,40]. With the exception of one study [39], testing for respiratory viruses other than SARS-CoV-2 was carried out at physician discretion or restricted to patients admitted to critical care due to capacity limitations [16]. Consequently, bias towards testing those with severe disease is likely an important confounder. Le Hingrat *et al.* [39] tested all patients hospitalised with influenza like illness at a ‘COVID-19 first line hospital’ in Paris, France, between 25th January and 30th April 2020, for SARS-CoV-2 and a broad range of other respiratory viruses, and identified co-infection in 6% (49/806) of patients, but it was a single-centre study over a shorter time period.

Pre-pandemic viral co-infections were known to be more common in children [41], particularly the under 5s. 2.2% of our participants were under 18, but this group showed the highest prevalence of viral co-infection (4.5%). This suggests that a similar pattern may be seen in COVID-19 viral co-infection.

However, no significant patient factors, symptoms, or laboratory results were identified as associated with viral co-infection. This illustrates the inability to distinguish between mono-infected and co-infected patients clinically, and the need for broad syndromic respiratory viral testing.

Our study showed associations between previously identified risk factors for severe COVID-19 disease, such as male sex and increasing age [9], and admission to critical care or death, however we did not show such an association with co-infection. This may be due to the low prevalence of co-infected patients identified in our study.

The strength of our study comes from being a large multi-centre prospective study with comprehensive clinical metadata encompassing a broad study period. Additionally, we used a gold standard diagnostic test, that detects a broad range of endemic respiratory pathogens and is in routine use in the clinical setting, to test for co-infection in a systematic and unbiased manner.

Disentangling the effect of public health measures on virus circulation from potential direct interactions between viruses is difficult. Interactions between co-circulating respiratory viruses can influence patterns of infection at a population level [42]. For example, it has been suggested that the circulation of rhinovirus delayed the spread of pandemic H1N1 Influenza A virus in France in 2009 [43] whilst Nickbakhsh *et al* [42] demonstrated both positive and negative correlations in prevalence between different pairs of viruses even after key alternative drivers of infection such as patient age and seasonal variability were taken into account. Cellular interactions between respiratory viruses during co-infection are likely to result in interlinked patterns of infection at a population level. Several mechanisms of viral interference have been suggested, including the direct blockade of viral entry receptors, viral competition for host cell resources and the viral induction of immune responses that protect against infection by other viruses [44]. Replication of SARS-CoV-2 in human respiratory epithelial cells has been shown to be inhibited by co-infection with rhinovirus [45], and the interferon response induced by rhinovirus infection has been implicated in protecting against subsequent infection with influenza A [44].

Prior to COVID-19, viral co-infection had been implicated in exacerbating disease severity [2–6,46]. However, our study did not demonstrate a significant association between viral co-infection and COVID-19 severity. Although the systematic testing of patients in our study circumvented the selection bias reported in other studies towards increased propensity to test for other respiratory viruses in those with severe disease [38], the small numbers of participants with co-infections will have affected our ability to assess the impact of co-infection on disease severity and identify patient characteristics (risk factors) associated with co-infection..

Animal models of SARS-CoV-2 and influenza A co-infection have demonstrated enhanced disease severity [47,48], as have three large studies of influenza co-infection in COVID-19 patients albeit in cohorts that were not systematically tested for endemic respiratory viruses [15,16,38]. We did not detect any co-infection with influenza A in our cohort, thus could evaluate the impact of influenza co-infection on disease severity in COVID-19.

The unrestricted co-circulation of SARS-CoV-2 with other respiratory viruses is expected to result in a higher incidence of viral co-infections in COVID-19 patients. If co-infections are to become more frequent, then ongoing studies are needed to ascertain the optimal management for these patients. Corticosteroids are recommended in COVID-19 patients requiring supplemental oxygen [49]; a recent Cochrane review showed that corticosteroid treatment is associated with increased risk of mortality and hospital-acquired infection in patients with influenza infection [50], however, the best approach for co-infected patients is currently unknown.

Our study has a number of limitations. First, the small numbers of co-infections detected impacted our ability to identify statistical differences between co-infected and mono-infected groups, and between those co-infected with different viruses. There was heterogeneity in the interval between symptom onset and enrolment samples were taken, with a median of 9 days. This could have resulted in the misclassification of participants as having no co-infection if shedding of co-infecting virus had stopped prior to sampling. Sampling earlier in the course of infection would provide the most accurate measure of co-infection rates and identify true co-infection as opposed to secondary infection. Moreover, the absence of longitudinal samples precluded any analysis of the effect of the order in which infections were acquired. Lastly, our cohort of COVID-19 patients were recruited predominantly from the first wave of the pandemic. Only a small proportion of samples (8%, 83/1002) were collected between July 2020 and June 2021, which will have restricted our ability to detect co-infections during this period.

A low prevalence of viral co-infection was observed in hospitalised COVID-19 patients in the UK during the first 18 months of the pandemic, likely due to strict COVID mitigation measures. However, as these measures are relaxed, and variants continue to evolve, we are seeing a resurgence in endemic respiratory virus circulation. Ongoing respiratory viral surveillance is vital to characterise the interactions between SARS-CoV-2 and endemic respiratory viruses, such as influenza and RSV, and to ascertain the frequency of viral co-infection and its impact on disease severity and healthcare utilisation.

## Tables

### Table 1. Demographics, physiological and laboratory parameters at admission, and clinical outcomes for cohort of 1002 COVID-19 patients

| | *n* | | *All patients* | *Co-infected* | *Not co-infected* | *p-value*[a] |
|---|---|---|---|---|---|---|
| **Characteristics** | | | | | | |
| Sex | 1001 | Female | 379 (37.9) | 8 (40.0) | 371 (37.8) | 0.820 |

| | | | | | | |
|---|---|---|---|---|---|---|
| | | Male | 622 (62.1) | 12 (60.0) | 610 (62.2) | |
| Age (years) | 993 | < 18 | 22 (2.2) | 1 (5.0) | 21 (2.2) | 0.157 |
| | | 18 to 35 | 59 (5.9) | 2 (10.0) | 57 (5.9) | |
| | | 35 to 50 | 190 (19.1) | 1 (5.0) | 189 (19.4) | |
| | | 50 to 70 | 442 (44.5) | 12 (60.0) | 430 (44.2) | |
| | | >70 | 280 (28.2) | 4 (20.0) | 276 (28.4) | |
| | | Median (IQR) | 60.0 (49.0 to 72.0) | 61.5 (51.8 to 69.2) | 60.0 (49.0 to 72.0) | 0.893 |
| Ethnicity | 935 | White | 739 (79.0) | 17 (89.5) | 722 (78.8) | 0.945 |
| | | Black | 64 (6.8) | 1 (5.3) | 63 (6.9) | |
| | | South Asian | 55 (5.9) | 0 (0.0) | 55 (6.0) | |
| | | Other | 50 (5.3) | 1 (5.3) | 49 (5.3) | |
| | | East Asian | 18 (1.9) | 0 (0.0) | 18 (2.0) | |
| | | Arab | 6 (0.6) | 0 (0.0) | 6 (0.7) | |
| | | West Asian | 2 (0.2) | 0 (0.0) | 2 (0.2) | |
| | | Asian | 1 (0.1) | 0 (0.0) | 1 (0.1) | |
| Number of Comorbidities | 1002 | 0 | 406 (40.5) | 10 (50.0) | 396 (40.3) | 0.266 |
| | | 1 | 315 (31.4) | 3 (15.0) | 312 (31.8) | |
| | | 2+ | 281 (28.0) | 7 (35.0) | 274 (27.9) | |
| | | Median | 0.5 | 1.0 | 1.0 | 0.759 |

| | | | | | | |
|---|---|---|---|---|---|---|
| | | (IQR) | (0.0 to 2.0) | (0.0 to 2.0) | (0.0 to 2.0) | |
| Immunocompromise | 1002 | No | 833 (83.1) | 15 (75.0) | 818 (83.3) | 0.361 |
| | | Yes | 169 (16.9) | 5 (25.0) | 164 (16.7) | |
| **Observations** | | | | | | |
| Temperature (°C) | 938 | | 37.4<br>(36.7 to 38.2) | 37.6<br>(36.9 to 38.2) | 37.4<br>(36.7 to 38.2) | 0.559 |
| Heart Rate (beats/min) | 953 | | 92.0<br>(80.0 to 107.0) | 96.0<br>(80.5 to 103.5) | 92.0<br>(80.0 to 107.0) | 0.917 |
| Respiratory Rate (breaths/min) | 939 | | 22.0<br>(18.0 to 26.0) | 19.0<br>(18.0 to 28.0) | 22.0<br>(18.8 to 26.0) | 0.443 |
| **Laboratory Results** | | | | | | |
| White Cell Count (x$10^9$ cells/L) | 631 | | 7.2<br>(5.1 to 9.4) | 9.8<br>(5.5 to 11.2) | 7.1<br>(5.1 to 9.3) | 0.132 |
| Neutrophil Count (x$10^9$ cells/L) | 610 | | 5.3<br>(3.6 to 7.7) | 8.0<br>(4.1 to 9.9) | 5.3<br>(3.6 to 7.6) | 0.122 |
| Lymphocyte Count (x$10^9$ cells/L) | 610 | | 0.9<br>(0.7 to 1.3) | 1.0 (<br>0.8 to 1.2) | 0.9<br>(0.7 to 1.3) | 0.976 |
| CRP (mg/L) | 572 | | 92.0<br>(41.4 to 182.5) | 76.0<br>(41.0 to 226.0) | 93.0<br>(41.8 to 182.0) | 0.876 |
| **Radiology** | | | | | | |
| CXR Infiltrates | 409 | | 247 (60.4) | 6 (66.7) | 241 (60.2) | 1.000 |
| **Clinical Interventions and Outcomes** | | | | | | |
| Oxygen Required at Admission | 913 | | 354 (38.8) | 9 (47.4) | 345 (38.6) | 0.480 |
| Severity Score | 914 | | | | | 0.914 |

| | | | | | |
|---|---|---|---|---|---|
| 1 – no oxygen requirement | | 184 (20.1) | 3 (16.7) | 181 (20.2) | |
| 2 – supplementary oxygen only | | 256 (28.0) | 6 (33.3) | 250 (27.9) | |
| 3 – non-invasive ventilation/HFNO | | 169 (18.5) | 2 (11.1) | 167 (18.6) | |
| 4 – invasive ventilation | | 144 (15.8) | 3 (16.7) | 141 (15.7) | |
| 5 – death within 28 days | | 161 (17.6) | 4 (22.2) | 157 (17.5) | |
| | Median (IQR) | 2.5 (2.0 to 4.0) | 3.0 (2.0 to 4.0) | 3.0 (2.0 to 4.0) | 0.748 |
| Critical Care admission | 999 | 387 (38.7) | 9 (45.0) | 378 (38.6) | 0.644 |
| Death in hospital | 968 | 171 (17.7) | 4 (21.1) | 167 (17.6) | 0.760 |
| Length of stay in days (survivors) | 776 | 8.0 (5.0 to 16.0) | 10.0 (6.5 to 11.2) | 8.0 (5.0 to 16.0) | 0.688 |

Data are n(%) or median (IQR) unless otherwise stated

CRP, C-reactive protein; CXR, chest radiograph; HFNO, high flow nasal oxygen

[a] Fisher's exact test

### Table 2: Ordinal logistic regression analysis of risk factors for increasing COVID-19 disease severity score

| | *OR* | *2.5% CI* | *97.5% CI* | *P value* |
|---|---|---|---|---|
| Co-infection: Yes | 1.140 | 0.608 | 2.135 | p=0.681 |
| Sex: Male | 1.801 | 1.412 | 2.298 | p<0.001 |

| | | | | |
|---|---|---|---|---|
| Age (years) | 1.009 | 1.002 | 1.017 | p=0.017 |
| Number of Comorbidities: 1 | 1.433 | 1.149 | 1.788 | p=0.001 |
| Number of Comorbidities: 2+ | 0.908 | 0.741 | 1.114 | p=0.356 |
| Immunocompromised: Yes | 1.104 | 0.886 | 1.377 | p=0.376 |
| Timing of sample collection (days since collection of first patient sample) | 0.996 | 0.994 | 0.998 | p<0.001 |

## Figures

Figure 1: Study Design. a) Study flow chart. b) Location of study sites

Figure 2: a) Aetiology of co-infection; co-infecting viruses detected by multiplex PCR in cohort of 1002 hospitalised COVID-19 patients. b) Co-infection status of samples collected, by month. c) Co-infecting viruses by month of detection.

hRV, human rhinovirus; PIV-1-4, para-influenza viruses 1-4; Flu B, influenza B virus; hMPV, human metapneumovirus; RSV, respiratory syncytial virus.

Figure 3: Risk groups for co-infection. Multivariable logistic regression analysis adjusted odds plot.

Figure 4: Multivariable logistic regression analysis adjusted odds ratio plots. a) Risk factors for critical care admission. b) Risk factors for mortality.

### Funding

This work is supported by grants from the National Institute for Health Research (award CO-CIN-01), the Medical Research Council (grants MC_PC_19059 and MC_ST_CVR_2019), the National Institute for Health Research Health Protection Research Unit (NIHR HPRU) in Emerging and Zoonotic Infections at University of Liverpool (NIHR award 200907) and NIHR HPRU in Respiratory Infections at Imperial College London both in partnership with Public Health England (PHE) - now UK Health Security Agency (UK HAS) (NIHR award 200927), EU Platform for European Preparedness Against (Re-)emerging Epidemics, Wellcome Trust and Department for International Development (DID; 215091/Z/18/Z), and the Bill and Melinda Gates Foundation (OPP1209135). Liverpool Experimental Cancer Medicine Centre provided infrastructure support for this research (grant reference: C18616/A25153).

## Patient Consent Statement

Ethics approval for the study protocol and informed consent forms was given by the South Central-Oxford C Research Ethics Committee in England (13/SC/0149), the Scotland A Research Ethics Committee (20/SS/0028), and the WHO Ethics Review Committee (RPC571 and RPC572; April 2013). Patient's written informed consent was obtained for the collection of biological samples.

All study documents are available at https://isaric4c.net/protocols. The study is registered with ISRCTN https://www.isrctn.com/ISRCTN66726260.

## Acknowledgments

This work uses data provided by patients and collected by the NHS as part of their care and supports #DataSavesLives. We are extremely grateful to the 2648 frontline NHS clinical and research staff and volunteer medical students who collected this data in challenging circumstances and for the generosity of the participants and their families for their individual contributions in these difficult times.

## Potential Conflicts of Interest

SEM reports funding from the Medical Research Council (MRC). DP reports funding from UK Research and Innovation (UKRI) and payment from the University of Cambridge – Sir Isaac Newton Institute for conference organisation. EV reports funding from the MRC

(MC_ST_CVR_2019) and lecture fees from Janssen. AH reports grants from the MRC and Public Health Scotland, and was a member of the National Institute for Health Research (NIHR) Urgent Public Health Group. MGS reports grants from NIHR UK, MRC, and the Health Protection Research Unit (HPRU) in Emerging and Zoonotic Infections, University of Liverpool, during the conduct of the study. MGS is chair of the scientific advisory board and a minority shareholder at Integrum Scientific, and director and majority shareholder of MedEx Solutions Ltd, both unrelated to this submission. MGS is a non-remunerated member of Pfizer's External Data Monitoring Committee for their mRNA vaccine program(s), HMG UK Scientific Advisory Group for Emergencies (SAGE), COVID-19 Response, and HMG UK New Emerging Respiratory Virus Threats Advisory Group (NERVTAG). JKB reports grants from the UK Department of Health and Social Care (DHSC), the Wellcome Trust and MRC. PJMO reports personal fees from consultancies (ie, GlaxoSmithKline, Janssen, Bavarian Nordic, Pfizer, and Cepheid) and for the European Respiratory Society; grants from the MRC, MRC Global Challenge Research Fund, EU, NIHR Biomedical Research Centre, MRC–GlaxoSmithKline, Wellcome Trust, and NIHR (HPRU in Respiratory Infection); and is an NIHR senior investigator, unrelated to this submission. PJMO's role as president of the British Society for Immunology was unpaid, but travel and accommodation at some meetings were paid for by the society.

### †ISARIC4C investigators

**Co-Lead Investigator (Consortium Lead)**: J Kenneth Baillie.

**Co-Lead Investigator (Imperial College)*: Peter JM Openshaw.

**Co-Lead Investigator (Sponsor & Protocol CI)*: Malcolm G Semple.

*Co-Investigator*: Beatrice Alex, Petros Andrikopoulos, Benjamin Bach, Wendy S Barclay, Debby Bogaert, Meera Chand, Kanta Chechi, Graham S Cooke, Ana da Silva Filipe, Thushan de Silva,

Annemarie B Docherty, Gonçalo dos Santos Correia, Marc-Emmanuel Dumas, Jake Dunning, Tom Fletcher, Christoper A Green, William Greenhalf, Julian L Griffin, Rishi K Gupta, Ewen M Harrison, Julian A Hiscox, Antonia Ying Wai Ho, Karl Holden, Peter W Horby, Samreen Ijaz, Saye Khoo, Paul Klenerman, Andrew Law, Matthew R Lewis, Sonia Liggi, Wei Shen Lim, Lynn Maslen, Alexander J Mentzer, Laura Merson, Alison M Meynert, Shona C Moore, Mahdad Noursadeghi, Michael Olanipekun, Anthonia Osagie, Massimo Palmarini , Carlo Palmieri, William A Paxton, Georgios Pollakis, Nicholas Price, Andrew Rambaut, David L Robertson, Clark D Russell, Vanessa Sancho-Shimizu, Caroline J Sands, Janet T Scott, Louise Sigfrid, Tom Solomon, Shiranee Sriskandan, David Stuart, Charlotte Summers, Olivia V Swann, Zoltan Takats, Panteleimon Takis, Richard S Tedder, AA Roger Thompson, Emma C Thomson, Ryan S Thwaites, Lance CW Turtle, Maria Zambon.

*Data Analyst*: Thomas M Drake, Cameron J Fairfield, Stephen R Knight, Kenneth A Mclean, Derek Murphy, Lisa Norman , Riinu Pius, Catherine A Shaw.

*Data and Information System Manager*: Marie Connor, Jo Dalton, Carrol Gamble, Michelle Girvan, Sophie Halpin, Janet Harrison, Clare Jackson, James Lee, Laura Marsh, Daniel Plotkin, Stephanie Roberts, Egle Saviciute.

*Data Integration and Presentation*: Sara Clohisey, Ross Hendry, Susan Knight, Eva Lahnsteiner, Andrew Law, Gary Leeming, Lucy Norris, James Scott-Brown, Sarah Tait, Murray Wham.

*ISARIC Clinical Coordinator*: Gail Carson.

*Edinburgh Laboratory Staff and Volunteers*: Richard Clark, Audrey Coutts, Lorna Donnelly, Angie Fawkes, Tammy Gilchrist, Katarzyna Hafezi, Louise MacGillivray, Alan Maclean, Sarah McCafferty, Kirstie Morrice, Lee Murphy, Nicola Wrobel.

*Local Principal Investigators*: Kayode Adeniji, Daniel Agranoff, Ken Agwuh, Dhiraj Ail, Erin L. Aldera, Ana Alegria, Sam Allen, Brian Angus, Abdul Ashish, Dougal Atkinson, Shahedal Bari,

Gavin Barlow, Stella Barnass, Nicholas Barrett, Christopher Bassford, Sneha Basude, David Baxter, Michael Beadsworth, Jolanta Bernatoniene, John Berridge, Colin Berry, Nicola Best, Pieter Bothma, Robin Brittain-Long, Naomi Bulteel, Tom Burden, Andrew Burtenshaw, Vikki Caruth, David Chadwick, David Chadwick, Duncan Chambler, Nigel Chee, Jenny Child, Srikanth Chukkambotla, Tom Clark, Paul Collini, Catherine Cosgrove, Jason Cupitt, Maria-Teresa Cutino-Moguel, Paul Dark, Chris Dawson, Samir Dervisevic, Phil Donnison, Sam Douthwaite, Andrew Drummond, Ingrid DuRand, Ahilanadan Dushianthan, Tristan Dyer, Cariad Evans, Chi Eziefula, Chrisopher Fegan, Adam Finn, Duncan Fullerton, Sanjeev Garg, Sanjeev Garg, Atul Garg, Effrossyni Gkrania-Klotsas, Jo Godden, Arthur Goldsmith, Clive Graham, Tassos Grammatikopoulos, Elaine Hardy, Stuart Hartshorn, Daniel Harvey, Peter Havalda, Daniel B Hawcutt, Maria Hobrok, Luke Hodgson, Anil Hormis, Joanne Howard, Michael Jacobs, Susan Jain, Paul Jennings, Agilan Kaliappan, Vidya Kasipandian, Stephen Kegg, Michael Kelsey, Jason Kendall, Caroline Kerrison, Ian Kerslake, Oliver Koch, Gouri Koduri, George Koshy, Shondipon Laha, Steven Laird, Susan Larkin, Tamas Leiner, Patrick Lillie, James Limb, Vanessa Linnett, Jeff Little, Mark Lyttle, Michael MacMahon, Emily MacNaughton, Ravish Mankregod, Huw Masson, Elijah Matovu, Katherine McCullough, Ruth McEwen, Manjula Meda, Gary Mills, Jane Minton, Mariyam Mirfenderesky, Kavya Mohandas, Quen Mok, James Moon, Elinoor Moore, Patrick Morgan, Craig Morris, Katherine Mortimore, Samuel Moses, Mbiye Mpenge, Rohinton Mulla, Michael Murphy, Thapas Nagarajan, Megan Nagel, Mark Nelson, Lillian Norris, Matthew K. O'Shea, Marlies Ostermann, Igor Otahal, Mark Pais, Carlo Palmieri, Selva Panchatsharam, Danai Papakonstantinou, Padmasayee Papineni, Hassan Paraiso, Brij Patel, Natalie Pattison, Justin Pepperell, Mark Peters, Mandeep Phull, Stefania Pintus, Tim Planche, Frank Post, David Price, Rachel Prout, Nikolas Rae, Henrik Reschreiter, Tim Reynolds, Neil Richardson, Mark Roberts, Devender Roberts, Alistair Rose, Guy Rousseau, Bobby Ruge, Brendan Ryan, Taranprit Saluja, Sarah Cole, Matthias L Schmid, Aarti Shah, Manu Shankar-Hari, Prad Shanmuga, Anil Sharma, Anna Shawcross, Jagtur Singh Pooni, Jeremy Sizer, Richard Smith, Catherine Snelson, Nick

Spittle, Nikki Staines, Tom Stambach, Richard Stewart, Pradeep Subudhi, Tamas Szakmany, Kate Tatham, Jo Thomas, Chris Thompson, Robert Thompson, Ascanio Tridente, Darell Tupper-Carey, Mary Twagira, Nick Vallotton, Rama Vancheeswaran, Lisa Vincent-Smith, Shico Visuvanathan, Alan Vuylsteke, Sam Waddy, Rachel Wake, Andrew Walden, Ingeborg Welters, Tony Whitehouse, Paul Whittaker, Ashley Whittington, Meme Wijesinghe, Martin Williams, Lawrence Wilson, Stephen Winchester, Martin Wiselka, Adam Wolverson, Daniel G Wootton, Andrew Workman, Bryan Yates, Peter Young.

*Material Management*: Sarah E McDonald, Victoria Shaw.

*Outbreak Laboratory Staff and Volunteers*: Katie A. Ahmed, Jane A Armstrong, Milton Ashworth, Innocent G Asiimwe, Siddharth Bakshi, Samantha L Barlow, Laura Booth, Benjamin Brennan, Katie Bullock, Nicola Carlucci , Emily Cass, Benjamin WA Catterall, Jordan J Clark, Emily A Clarke, Sarah Cole, Louise Cooper, Helen Cox, Christopher Davis, Oslem Dincarslan, Alejandra Doce Carracedo, Chris Dunn, Philip Dyer, Angela Elliott, Anthony Evans, Lorna Finch, Lewis WS Fisher, Lisa Flaherty, Terry Foster, Isabel Garcia-Dorival, Philip Gunning, Catherine Hartley, Anthony Holmes, Rebecca L Jensen, Christopher B Jones, Trevor R Jones, Shadia Khandaker, Katharine King, Robyn T. Kiy, Chrysa Koukorava, Annette Lake, Suzannah Lant, Diane Latawiec, Lara Lavelle-Langham, Daniella Lefteri, Lauren Lett, Lucia A Livoti, Maria Mancini, Hannah Massey, Nicole Maziere, Sarah McDonald, Laurence McEvoy, John McLauchlan, Soeren Metelmann, Nahida S Miah, Joanna Middleton, Joyce Mitchell, Shona C Moore, Ellen G Murphy, Rebekah Penrice-Randal, Jack Pilgrim, Tessa Prince, Will Reynolds, P. Matthew Ridley, Debby Sales, Victoria E Shaw, Rebecca K Shears, Benjamin Small, Krishanthi S Subramaniam, Agnieska Szemiel, Aislynn Taggart, Jolanta Tanianis-Hughes, Jordan Thomas, Erwan Trochu, Libby van Tonder, Eve Wilcock, J. Eunice Zhang.

*Patient Engagement*: Seán Keating.

*Project Administrator*: Cara Donegan, Rebecca G. Spencer.

*Project Manager*: Chloe Donohue, Fiona Griffiths, Hayley Hardwick, Wilna Oosthuyzen.

### Data sharing

Access to all data and samples collected by ISARIC4C are controlled by an Independent Data and Materials Access Committee comprising representatives of research funders, academia, clinical medicine, public health, and industry. The application process for access to the data is available on the ISARIC4C website.

### Dissemination

Dissemination to participants and related patient and public communities: ISARIC4C has a public facing website and twitter account (@CCPUKstudy). We are engaging with print and internet press, television, radio, news, and documentary programme makers. We will explore distribution of findings with The Asthma UK and British Lung Foundation Partnership and take advice from NIHR Involve and GenerationR Alliance Young People's Advisory Groups.

### Data Presentation

Preliminary results from this study were presented at the European Congress for Clinical Microbiology and Infectious Diseases (ECCMID) in Lisbon, Portugal on 25th April 2022. Scottish data was presented at the European Scientific Working Group on Influenza and other Respiratory Viruses (ESWI) Conference online on 5th December 2021.

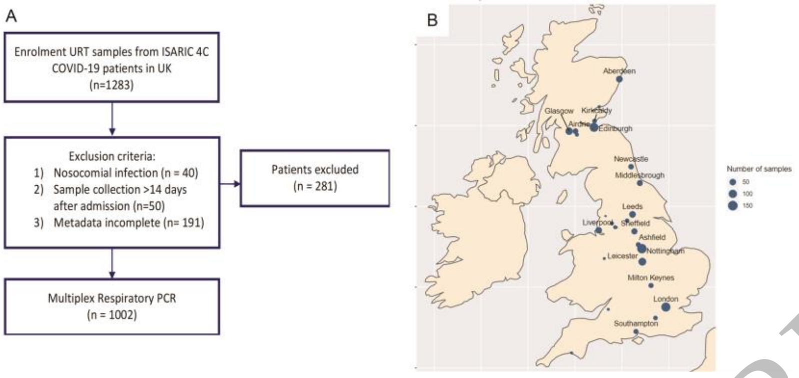

*Figure 1*
*106x49 mm ( x DPI)*

*Figure 2*
*450x144 mm ( x DPI)*

*Figure 3*
*289x166 mm ( x DPI)*

*Figure 4*

*281x170 mm ( x DPI)*

## Supplementary Material

ofac531_Supplementary_Data

