# Viral Coinfections in Hospitalized Coronavirus Disease 2019 Patients Recruited to the International Severe Acute Respiratory and Emerging Infections Consortium WHO Clinical Characterisation Protocol UK Study

**DOI:** 10.1093/ofid/ofac531

**Published:** 2022-10-10

**Authors:** Elen Vink, Chris Davis, Alasdair MacLean, David Pascall, Sarah E McDonald, Rory Gunson, Hayley E Hardwick, Wilna Oosthuyzen, Peter J M Openshaw, J Kenneth Baillie, Malcolm G Semple, Antonia Ho, ISARIC4C Investigators, J Kenneth Baillie, Peter J M Openshaw, Malcolm G Semple, Wendy S Barclay, Debby Bogaert, Meera Chand, Kanta Chechi, Graham S Cooke, Ana da Silva Filipe, Thushan de Silva, Annemarie B Docherty, Gonçalo dos Santos Correia, Marc-Emmanuel Dumas, Jake Dunning, Tom Fletcher, Christoper A Green, William Greenhalf, Julian L Griffin, Rishi K Gupta, Ewen M Harrison, Julian A Hiscox, Antonia Ying Wai Ho, Karl Holden, Peter W Horby, Samreen Ijaz, Saye Khoo, Paul Klenerman, Andrew Law, Matthew R Lewis, Sonia Liggi, Wei Shen Lim, Lynn Maslen, Alexander J Mentzer, Laura Merson, Alison M Meynert, Shona C Moore, Mahdad Noursadeghi, Michael Olanipekun, Anthonia Osagie, Massimo Palmarini, Carlo Palmieri, William A Paxton, Georgios Pollakis, Nicholas Price, Andrew Rambaut, David L Robertson, Clark D Russell, Vanessa Sancho-Shimizu, Caroline J Sands, Janet T Scott, Louise Sigfrid, Tom Solomon, Shiranee Sriskandan, David Stuart, Charlotte Summers, Olivia V Swann, Zoltan Takats, Panteleimon Takis, Richard S Tedder, A A Roger Thompson, Emma C Thomson, Ryan S Thwaites, Lance CW Turtle, Maria Zambon, Thomas M Drake, Cameron J Fairfield, Stephen R Knight, Kenneth A Mclean, Derek Murphy, Lisa Norman, Riinu Pius, Catherine A Shaw, Marie Connor, Jo Dalton, Carrol Gamble, Michelle Girvan, Sophie Halpin, Janet Harrison, Clare Jackson, James Lee, Laura Marsh, Daniel Plotkin, Stephanie Roberts, Egle Saviciute, Sara Clohisey, Ross Hendry, Susan Knight, Eva Lahnsteiner, Andrew Law, Gary Leeming, Lucy Norris, James Scott-Brown, Sarah Tait, Murray Wham, Richard Clark, Audrey Coutts, Lorna Donnelly, Angie Fawkes, Tammy Gilchrist, Katarzyna Hafezi, Louise MacGillivray, Alan Maclean, Sarah McCafferty, Kirstie Morrice, Lee Murphy, Nicola Wrobel, Kayode Adeniji, Daniel Agranoff, Ken Agwuh, Dhiraj Ail, Erin L Aldera, Ana Alegria, Sam Allen, Brian Angus, Abdul Ashish, Dougal Atkinson, Shahedal Bari, Gavin Barlow, Stella Barnass, Nicholas Barrett, Christopher Bassford, Sneha Basude, David Baxter, Michael Beadsworth, Jolanta Bernatoniene, John Berridge, Colin Berry, Nicola Best, Pieter Bothma, Robin Brittain-Long, Naomi Bulteel, Tom Burden, Andrew Burtenshaw, Vikki Caruth, David Chadwick, David Chadwick, Duncan Chambler, Nigel Chee, Jenny Child, Srikanth Chukkambotla, Tom Clark, Paul Collini, Catherine Cosgrove, Jason Cupitt, Maria-Teresa Cutino-Moguel, Paul Dark, Chris Dawson, Samir Dervisevic, Phil Donnison, Sam Douthwaite, Andrew Drummond, Ingrid DuRand, Ahilanadan Dushianthan, Tristan Dyer, Cariad Evans, Chi Eziefula, Chrisopher Fegan, Adam Finn, Duncan Fullerton, Sanjeev Garg, Sanjeev Garg, Atul Garg, Effrossyni Gkrania-Klotsas, Jo Godden, Arthur Goldsmith, Clive Graham, Tassos Grammatikopoulos, Elaine Hardy, Stuart Hartshorn, Daniel Harvey, Peter Havalda, Daniel B Hawcutt, Maria Hobrok, Luke Hodgson, Anil Hormis, Joanne Howard, Michael Jacobs, Susan Jain, Paul Jennings, Agilan Kaliappan, Vidya Kasipandian, Stephen Kegg, Michael Kelsey, Jason Kendall, Caroline Kerrison, Ian Kerslake, Oliver Koch, Gouri Koduri, George Koshy, Shondipon Laha, Steven Laird, Susan Larkin, Tamas Leiner, Patrick Lillie, James Limb, Vanessa Linnett, Jeff Little, Mark Lyttle, Michael MacMahon, Emily MacNaughton, Ravish Mankregod, Huw Masson, Elijah Matovu, Katherine McCullough, Ruth McEwen, Manjula Meda, Gary Mills, Jane Minton, Mariyam Mirfenderesky, Kavya Mohandas, Quen Mok, James Moon, Elinoor Moore, Patrick Morgan, Craig Morris, Katherine Mortimore, Samuel Moses, Mbiye Mpenge, Rohinton Mulla, Michael Murphy, Thapas Nagarajan, Megan Nagel, Mark Nelson, Lillian Norris, Matthew K O'Shea, Marlies Ostermann, Igor Otahal, Mark Pais, Carlo Palmieri, Selva Panchatsharam, Danai Papakonstantinou, Padmasayee Papineni, Hassan Paraiso, Brij Patel, Natalie Pattison, Justin Pepperell, Mark Peters, Mandeep Phull, Stefania Pintus, Tim Planche, Frank Post, David Price, Rachel Prout, Nikolas Rae, Henrik Reschreiter, Tim Reynolds, Neil Richardson, Mark Roberts, Devender Roberts, Alistair Rose, Guy Rousseau, Bobby Ruge, Brendan Ryan, Taranprit Saluja, Sarah Cole, Matthias L Schmid, Aarti Shah, Manu Shankar-Hari, Prad Shanmuga, Anil Sharma, Anna Shawcross, Jagtur Singh Pooni, Jeremy Sizer, Richard Smith, Catherine Snelson, Nick Spittle, Nikki Staines, Tom Stambach, Richard Stewart, Pradeep Subudhi, Tamas Szakmany, Kate Tatham, Jo Thomas, Chris Thompson, Robert Thompson, Ascanio Tridente, Darell Tupper-Carey, Mary Twagira, Nick Vallotton, Rama Vancheeswaran, Lisa Vincent-Smith, Shico Visuvanathan, Alan Vuylsteke, Sam Waddy, Rachel Wake, Andrew Walden, Ingeborg Welters, Tony Whitehouse, Paul Whittaker, Ashley Whittington, Meme Wijesinghe, Martin Williams, Lawrence Wilson, Stephen Winchester, Martin Wiselka, Adam Wolverson, Daniel G Wootton, Andrew Workman, Bryan Yates, Peter Young, Sarah E McDonald, Victoria Shaw, Katie A Ahmed, Jane A Armstrong, Milton Ashworth, Innocent G Asiimwe, Siddharth Bakshi, Samantha L Barlow, Laura Booth, Benjamin Brennan, Katie Bullock, Nicola Carlucci, Emily Cass, Benjamin W A Catterall, Jordan J Clark, Emily A Clarke, Sarah Cole, Louise Cooper, Helen Cox, Christopher Davis, Oslem Dincarslan, Alejandra Doce Carracedo, Chris Dunn, Philip Dyer, Angela Elliott, Anthony Evans, Lorna Finch, Lewis W S Fisher, Lisa Flaherty, Terry Foster, Isabel Garcia-Dorival, Philip Gunning, Catherine Hartley, Anthony Holmes, Rebecca L Jensen, Christopher B Jones, Trevor R Jones, Shadia Khandaker, Katharine King, Robyn T Kiy, Chrysa Koukorava, Annette Lake, Suzannah Lant, Diane Latawiec, Lara Lavelle-Langham, Daniella Lefteri, Lauren Lett, Lucia A Livoti, Maria Mancini, Hannah Massey, Nicole Maziere, Sarah McDonald, Laurence McEvoy, John McLauchlan, Soeren Metelmann, Nahida S Miah, Joanna Middleton, Joyce Mitchell, Shona C Moore, Ellen G Murphy, Rebekah Penrice-Randal, Jack Pilgrim, Tessa Prince, Will Reynolds, P Matthew Ridley, Debby Sales, Victoria E Shaw, Rebecca K Shears, Benjamin Small, Krishanthi S Subramaniam, Agnieska Szemiel, Aislynn Taggart, Jolanta Tanianis-Hughes, Jordan Thomas, Erwan Trochu, Libby van Tonder, Eve Wilcock, J Eunice Zhang, Seán Keating, Cara Donegan, Rebecca G Spencer, Chloe Donohue, Fiona Griffiths, Hayley Hardwick, Wilna Oosthuyzen

**Affiliations:** Medical Research Council-University of Glasgow Centre for Virus Research, University of Glasgow, Glasgow, United Kingdom; Medical Research Council-University of Glasgow Centre for Virus Research, University of Glasgow, Glasgow, United Kingdom; West of Scotland Specialist Virology Centre, NHS Greater Glasgow and Clyde, Glasgow, United Kingdom; MRC Biostatistics Unit, University of Cambridge, Cambridge, United Kingdom; Joint Universities Pandemic and Epidemiological Research (JUNIPER) Consortium; Medical Research Council-University of Glasgow Centre for Virus Research, University of Glasgow, Glasgow, United Kingdom; West of Scotland Specialist Virology Centre, NHS Greater Glasgow and Clyde, Glasgow, United Kingdom; NIHR Health Protection Research Unit in Emerging and Zoonotic Infections, Institute of Infection, Veterinary and Ecological Sciences, Faculty of Health and Life Sciences, University of Liverpool, Liverpool, United Kingdom; Roslin Institute, University of Edinburgh, Edinburgh, United Kingdom; National Heart and Lung Institute, Imperial College London, London, United Kingdom; Roslin Institute, University of Edinburgh, Edinburgh, United Kingdom; NIHR Health Protection Research Unit in Emerging and Zoonotic Infections, Institute of Infection, Veterinary and Ecological Sciences, Faculty of Health and Life Sciences, University of Liverpool, Liverpool, United Kingdom; The Pandemic Institute, University of Liverpool, Liverpool, United Kingdom; Medical Research Council-University of Glasgow Centre for Virus Research, University of Glasgow, Glasgow, United Kingdom

**Keywords:** disease severity, PCR, respiratory virus, rhinovirus, SARS-CoV-2, surveillance

## Abstract

**Background:**

We conducted this study to assess the prevalence of viral coinfection in a well characterized cohort of hospitalized coronavirus disease 2019 (COVID-19) patients and to investigate the impact of coinfection on disease severity.

**Methods:**

Multiplex real-time polymerase chain reaction testing for endemic respiratory viruses was performed on upper respiratory tract samples from 1002 patients with COVID-19, aged <1 year to 102 years old, recruited to the International Severe Acute Respiratory and Emerging Infections Consortium WHO Clinical Characterisation Protocol UK study. Comprehensive demographic, clinical, and outcome data were collected prospectively up to 28 days post discharge.

**Results:**

A coinfecting virus was detected in 20 (2.0%) participants. Multivariable analysis revealed no significant risk factors for coinfection, although this may be due to rarity of coinfection. Likewise, ordinal logistic regression analysis did not demonstrate a significant association between coinfection and increased disease severity.

**Conclusions:**

Viral coinfection was rare among hospitalized COVID-19 patients in the United Kingdom during the first 18 months of the pandemic. With unbiased prospective sampling, we found no evidence of an association between viral coinfection and disease severity. Public health interventions disrupted normal seasonal transmission of respiratory viruses; relaxation of these measures mean it will be important to monitor the prevalence and impact of respiratory viral coinfections going forward.

## Teaser

In a multi-centre prospective cohort study of viral co-infection in hospitalised COVID-19 patients using unbiased systematic PCR testing, co-infection was rare during the first 18 months of the pandemic, with no observed association between viral co-infection and disease severity.

Severe acute respiratory syndrome coronavirus 2 (SARS-CoV-2) emerged as a new viral pathogen in China at the end of 2019 and has since become pandemic with over 626 million confirmed cases of the associated disease, coronavirus disease 2019 (COVID-19), and approximately 6.5 million deaths worldwide (up to October 27, 2022) [1]. Evidence from existing studies of patients with influenza, respiratory syncytial virus (RSV), and rhinovirus infections suggest that viral coinfections are associated with increased disease severity [2–6]. However, data on the frequency of coinfection in patients with COVID-19 and how coinfections impact on disease severity are scarce and variable. Few studies have systematically and prospectively tested for a broad range of respiratory viruses in a patient cohort with comprehensive clinical metadata to allow for analysis of the impact of viral coinfection on disease severity and clinical outcomes.

Coronavirus disease 2019 public health measures such as physical distancing, lockdowns, and travel restrictions have resulted in a dramatic reduction in the circulation of other respiratory viruses over the past 2 years [7, 8]. However, as countries across the world relax restrictions, community transmission of these viruses will likely rise, as will the risk of viral coinfections in patients with COVID-19. Understanding the nature, frequency, and impact of coinfections are key to optimize the clinical management of patients, inform vaccination strategies and best practice in infection prevention and control, in addition to guiding healthcare service planning and funding.

In this prospective study, we assessed the prevalence of viral coinfection in a well characterized cohort of hospitalized patients with COVID-19 in the United Kingdom (UK) by systematically testing upper respiratory tract (URT) samples for a broad range of respiratory viruses, and we investigated the impact of coinfection on disease severity and clinical outcomes using comprehensive clinical metadata.

## METHODS

### Study Setting and Approvals

The International Severe Acute Respiratory and Emerging Infections Consortium (ISARIC) WHO Clinical Characterisation Protocol UK (CCP-UK) study is a prospective cohort study that recruited inpatients from hospitals across the UK (National Institute for Health Research Clinical Research Ne2rk Central Portfolio Management System ID 14152) and was conducted by the ISARIC Coronavirus Clinical Characterisation Consortium (ISARIC4C). Ethics approval was given by the South Central-Oxford C Research Ethics Committee in England (13/SC/0149), the Scotland A Research Ethics Committee (20/SS/0028), and the WHO Ethics Review Committee (RPC571 and RPC572; April 2013). The study protocol and further details are available online [9].

### Study Participants

**Figure 1.**
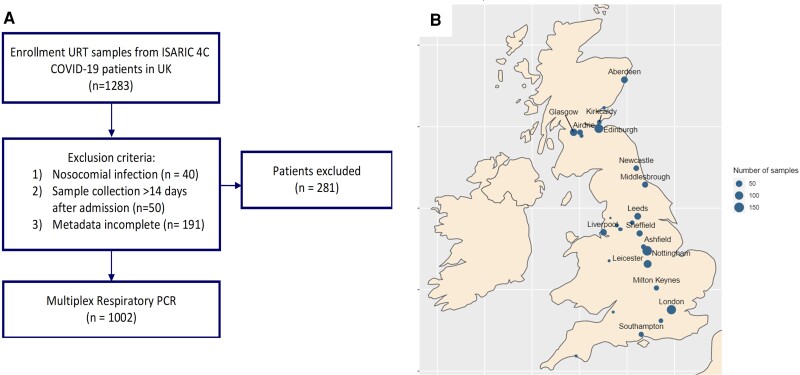
Study Design. (*A*) Study flow chart. (*B*) Location of study sites. COVID-19, coronavirus disease 2019; PCR, polymerase chain reaction; UK, United Kingdom; URT, upper respiratory tract.

Recruitment of COVID-19 cases to the ISARIC WHO CCP-UK study took place between February 5, 2020 and February 28, 2022 and has previously been described in detail [9]. In brief, demographic, clinical, laboratory, therapeutic, and outcome data were collected for participants with laboratory-confirmed or highly suspected COVID-19 on a standardized case report form. Data were uploaded to a Research Electronic Data Capture Database ([REDCap] Vanderbilt University, Nashville, TN; hosted by University of Oxford, Oxford, UK) [10]. A subset of ∼2500 participants gave consent for the collection of biological samples, including URT swabs, on days 1, 3, 9, and 28 postenrollment. The URT samples from all time points were not available for every participant due to the need to prioritize clinical care. We included day 1 (or if this was not available, day 3) URT samples only, and we excluded participants if their day 1 or day 3 samples were collected more than 14 days after admission or if the participant had nosocomial COVID-19, defined as symptom onset more than 7 days after admission (Figure 1). Participants were also excluded who were SARS-CoV-2 PCR negative.

### Collection and Ribonucleic Acid Extraction of Upper Respiratory Tract Swabs

Samples were collected from study participants using throat or nasal swabs that were placed into viral transport medium and stored at −80°C. Upper respiratory tract samples were inactivated and lysed with NucliSENS Lysis Buffer. Equine arteritis virus was used as an internal control (IC), which was introduced into the lysis buffer at this stage. Ribonucleic acid (RNA) extraction was performed using the fully automated Biomérieux EMAG platform with negative controls included on each extraction run. Eluates were stored at −80°C before testing.

### Multiplex Respiratory Polymerase Chain Reaction Panel

A validated in-house multiplex real-time polymerase chain reaction (PCR) panel for respiratory viruses, developed at the West of Scotland Specialist Virology Centre [11], was used to test 6 µL of extracted RNA for adenovirus, rhinovirus, RSV A and B, influenza A and B viruses, parainfluenza viruses 1–4, human coronaviruses (hCoV) NL63, 229E, and OC43, and human metapneumovirus. Positive controls for all viral targets, and negative controls were included in all PCR runs. Reverse transcription (RT)-PCR was performed on an Applied Biosystems 7500 Fast Real-Time PCR System. The following thermal profile was used: a single cycle of RT for 10 minutes at 50°C, 2 minutes at 95°C for RT inactivation and deoxyribonucleic acid polymerase activation, followed by 40 amplification cycles of 8 seconds at 95°C and 30 seconds at 60°C each (annealing-extension step). Data acquisition occurred at the annealing step of each cycle, and the threshold cycle (Ct) for each sample was calculated by determining the point at which the fluorescence exceeded the threshold limit. A Ct of <40 was considered positive.

### Coronavirus Disease 2019 Disease Severity Score

Participants were assigned a disease severity score according to maximum level of respiratory support required and clinical outcome. Categories were based on the World Health Organization (WHO) COVID-19 ordinal scale [12] but restricted to hospitalized patients: (1) no oxygen requirement (WHO score 3); (2) patient requiring oxygen by face mask or nasal prongs (WHO score 4); (3) patient requiring high-flow nasal oxygen or noninvasive ventilation (WHO score 5); (4) patients requiring mechanical ventilation (WHO score 6/7); and (5) patients who died within 28 days of admission (WHO score 8).

### Statistical Analysis

Categorical variables are reported as frequencies and percentages. Continuous variables are reported as means and standard deviations or medians and interquartile range (IQRs) based on normality. We investigated the association of demographic factors (age, sex, ethnicity, number of comorbidities, and immune status), clinical characteristics at admission (symptoms, observations, laboratory results, and radiology results), disease severity score, and clinical outcomes (admission to critical care, death with 28 days, and length of stay for survivors), with coinfection status using Fisher's exact test or Kruskal Wallis test, for categorical and continuous non-parametric variables, respectively. *P* ≤ .05 was considered statistically significant. Immunocompromise was classified as clinician defined or those receiving immunosuppressive therapy before hospital admission. Comorbidities were defined as those listed in the 4C Mortality Score [13].

Multivariable logistic regression analyses were used to seek associations between coinfection status and critical care admission or death, adjusting for the following variables (chosen a priori): age, sex, number of comorbidities, immune status, and timepoint within the study. Timepoint was defined as difference in days between the date of sample collection and the date of the earliest sample in the study, that is, February 5, 2020. Ordinal logistic regression analysis was used to investigate any association between coinfection status and disease severity score. All analyses were performed using R version 4.1.1 using the *Tidyverse*, *finalfit*, and *MASS* packages with model diagnostics performed using *PResiduals* and *sure* packages.

### Patient Consent Statement

Ethics approval for the study protocol and informed consent forms was given by the South Central-Oxford C Research Ethics Committee in England (13/SC/0149), the Scotland A Research Ethics Committee (20/SS/0028), and the WHO Ethics Review Committee (RPC571 and RPC572; April 2013). Patient's written informed consent was obtained for the collection of biological samples. All study documents are available at https://isaric4c.net/protocols. The study is registered with ISRCTN https://www.isrctn.com/ISRCTN66726260.

## RESULTS

This analysis included URT samples from 1002 participants admitted to 36 UK hospitals between February 5, 2020 and June 11, 2021 (Figure 1). The median participant age was 60 years (IQR, 49–72) and 622 (62.1%) were male (Table 1). Twenty-two (2.2%) participants were under 18 years of age. Five hundred ninety-six (59.4%) participants had 1 or more comorbidities (Table 1); the commonest comorbidities being diabetes (24.2%) and chronic cardiac disease (19.3%). One hundred sixty-nine participants (16.9%) were immunocompromised either due to underlying disease or immunosuppressant medication (Table 1).

**Table 1.** Demographics, Physiological, and Laboratory Parameters at Admission, and Clinical Outcomes for Cohort of 1002 COVID-19 Patients

Characteristics	n	All Patients	Coinfected	Not Coinfected	P Value^a^
Sex - Female	1001	379 (37.9)	8 (40.0)	371 (37.8)	.820
- Male		622 (62.1)	12 (60.0)	610 (62.2)	
Age (years) - <18	993	22 (2.2)	1 (5.0)	21 (2.2)	.157
- 18-35		59 (5.9)	2 (10.0)	57 (5.9)	
- 36-50		190 (19.1)	1 (5.0)	189 (19.4)	
- 51-70		442 (44.5)	12 (60.0)	430 (44.2)	
- >70		280 (28.2)	4 (20.0)	276 (28.4)	
Median (IQR)		60.0 (49.0 to 72.0)	61.5 (51.8 to 69.2)	60.0 (49.0 to 72.0)	.893
Ethnicity - White	935	739 (79.0)	17 (89.5)	722 (78.8)	.945
- Black		64 (6.8)	1 (5.3)	63 (6.9)	
- South Asian		55 (5.9)	0 (0.0)	55 (6.0)	
- Other		50 (5.3)	1 (5.3)	49 (5.3)	
- East Asian		18 (1.9)	0 (0.0)	18 (2.0)	
- Arab		6 (0.6)	0 (0.0)	6 (0.7)	
- West Asian		2 (0.2)	0 (0.0)	2 (0.2)	
- Asian		1 (0.1)	0 (0.0)	1 (0.1)	
Number of Comorbidities - 0	1002	406 (40.5)	10 (50.0)	396 (40.3)	.266
- 1		315 (31.4)	3 (15.0)	312 (31.8)	
- >2		281 (28.0)	7 (35.0)	274 (27.9)	
Median (IQR)		0.5 (0.0 to 2.0)	1.0 (0.0 to 2.0)	1.0 (0.0 to 2.0)	.759
Immunocompromise - No	1002	833 (83.1)	15 (75.0)	818 (83.3)	.361
- Yes		169 (16.9)	5 (25.0)	164 (16.7)	
Observations					
Temperature (°C)	938	37.4 (36.7 to 38.2)	37.6 (36.9 to 38.2)	37.4 (36.7 to 38.2)	.559
Heart rate (beats/min)	953	92.0 (80.0 to 107.0)	96.0 (80.5 to 103.5)	92.0 (80.0 to 107.0)	.917
Respiratory rate (breaths/min)	939	22.0 (18.0 to 26.0)	19.0 (18.0 to 28.0)	22.0 (18.8 to 26.0)	.443
Laboratory Results					
White cell count (×10^9^ cells/L)	631	7.2 (5.1 to 9.4)	9.8 (5.5 to 11.2)	7.1 (5.1 to 9.3)	.132
Neutrophil count (×10^9^ cells/L)	610	5.3 (3.6 to 7.7)	8.0 (4.1 to 9.9)	5.3 (3.6 to 7.6)	.122
Lymphocyte count (×10^9^ cells/L)	610	0.9 (0.7 to 1.3)	1.0 (0.8 to 1.2)	0.9 (0.7 to 1.3)	.976
CRP (mg/L)	572	92.0 (41.4 to 182.5)	76.0 (41.0 to 226.0)	93.0 (41.8 to 182.0)	.876
Radiology					
CXR infiltrates	409	247 (60.4)	6 (66.7)	241 (60.2)	1.000
Clinical Interventions and Outcomes					
Oxygen required at admission	913	354 (38.8)	9 (47.4)	345 (38.6)	.480
Severity score	914		…	…	.914
1—no oxygen requirement		184 (20.1)	3 (16.7)	181 (20.2)	
2—supplementary oxygen only		256 (28.0)	6 (33.3)	250 (27.9)	
3—noninvasive ventilation/HFNO		169 (18.5)	2 (11.1)	167 (18.6)	
4—invasive ventilation		144 (15.8)	3 (16.7)	141 (15.7)	
5—death within 28 days		161 (17.6)	4 (22.2)	157 (17.5)	
Median (IQR)		2.5 (2.0 to 4.0)	3.0 (2.0 to 4.0)	3.0 (2.0 to 4.0)	.748
Critical care admission	999	387 (38.7)	9 (45.0)	378 (38.6)	.644
Death in hospital	968	171 (17.7)	4 (21.1)	167 (17.6)	.760
Length of stay in days (survivors)	776	8.0 (5.0 to 16.0)	10.0 (6.5 to 11.2)	8.0 (5.0 to 16.0)	.688

Abbreviations: COVID-19, coronavirus disease 2019; CRP, C-reactive protein; CXR, chest radiograph; HFNO, high-flow nasal oxygen; IQR, interquartile range; min, minutes.

NOTE: Data are n (%) or median (IQR) unless otherwise stated.

a Fisher's exact test.

Median duration of symptoms at admission was 7 days (IQR, 3–10) (Supplementary Table 1); most frequently reported symptoms on admission were cough (79.2%), fever (76.0%), and shortness of breath (74.0%), and 60.4% (247 of 409) of admission chest x-rays revealed pulmonary infiltrates (Table 1). Median duration from symptom onset to sample collection was 9 days (IQR, 6–13). Three hundred eighty-seven (38.7%) participants were admitted to critical care with 263 (26.3%) participants requiring mechanical ventilation. Overall, 171 participants (16.9%) died and the median length of hospital admission for those discharged alive was 8 days (IQR, 5–16) (Table 1). All severity score groups were well represented in the patient cohort (Table 1).

**Figure 2.**
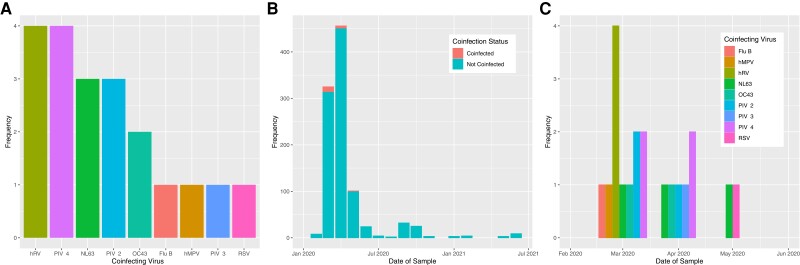
(*A*) Etiology of coinfection; coinfecting viruses detected by multiplex polymerase chain reaction in cohort of 1002 hospitalized COVID-19 patients. (*B*) Coinfection status of samples collected, by month. (*C*) Coinfecting viruses by month of detection. Flu B, influenza B virus; hMPV, human metapneumovirus; hRV, human rhinovirus; PIV-1–4, para-influenza viruses 1–4; RSV, respiratory syncytial virus.

A respiratory virus other than SARS-CoV-2 was detected in 20 (2.0%) patients with COVID-19. Rhinovirus and parainfluenza virus 4 were each identified in 4 (0.4%) participants, hCoV NL63 and parainfluenza virus 2 were identified in 3 participants (0.3%), hCoV OC43 was each identified in 2 participants, and influenza B virus, parainfluenza virus, human metapneumovirus, and RSV were detected in 1 participant each (Figure 2*A*). No cases of coinfection with more than 1 additional virus were detected. Participants with viral coinfection were all admitted before mid-May 2020 (Figure 2*B*). All cases of rhinovirus coinfection were detected during March 2020, and the diversity of coinfecting viruses diminished with time (Figure 2*C*).

**Figure 3.**
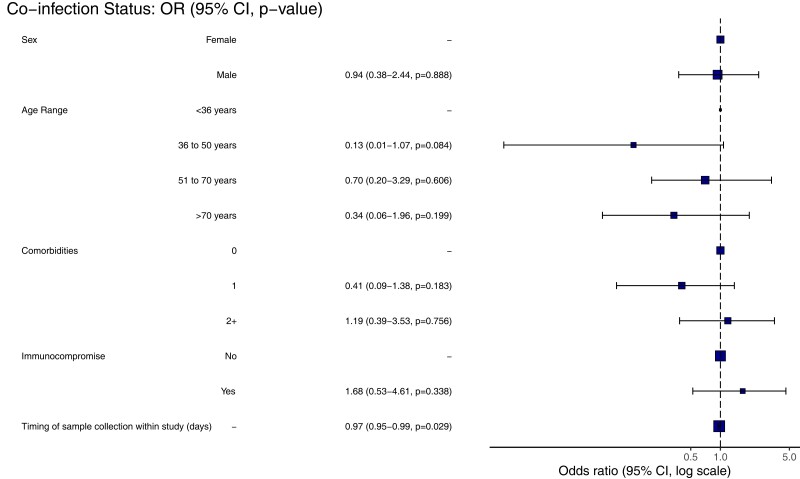
Risk groups for coinfection. Multivariable logistic regression analysis adjusted odds plot. CI, confidence interval; OR, odds ratio.

The highest proportion of coinfected participants was found in individuals under 18 years, with the lowest proportion in those aged 35–50 years, although these differences were not significant (Figure 3). Age (median 61.5 years [coinfected] vs 60.0 years [monoinfected], *P* = .89), sex (60.0% vs 62.2% male, *P* = .82), number of comorbidities (median 0.5 vs 1.0, *P* = .76), or immune status (25.0% vs 16.7% immunocompromised, *P* = .36) among COVID-19 patients did not differ by coinfection status (Table 1). Univariable and multivariable analysis demonstrated that the timing of SARS-CoV-2 infection within the pandemic was significantly associated with the risk of coinfection (adjusted odds ratio [aOR], 0.97; 95% confidence interval [CI], .95–.99; *P* = .03) (Figure 3) as would be expected given that all cases of coinfection were detected before mid-May 2020. No other significant risk factors for coinfection were identified (Figure 3).

Presenting symptoms and laboratory test results on admission did not differ significantly between monoinfected and coinfected participants. However, participants with viral coinfection reported wheeze more often (18.8% vs 7.8%, *P* = .13) (Supplementary Table 1) and had a higher median white cell count (9.8 vs 7.1 ×10^9^cells/L, *P* = .13), driven by a higher neutrophil count (8.0 vs 5.3 ×10^9^cells/L, *P* = .12) (Table 1). There was no observed difference in disease severity (median severity score 2.5 vs 3.0, *P* = .75), critical care admission (45.0% vs 38.6%, *P* = .64), death (21.0% vs 17.6%, *P* = .76), or the length of hospital admission for those discharged alive (8 vs 10 days, *P* = .69), between the 2 groups (Table 1).

**Figure 4.**
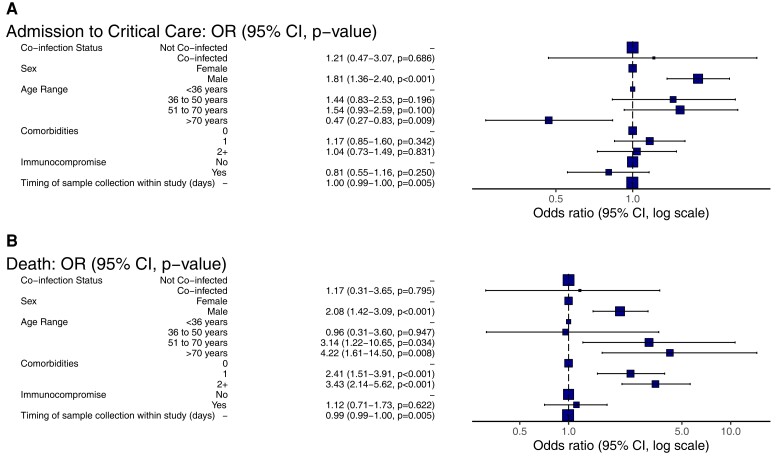
Multivariable logistic regression analysis adjusted odds ratio plots. (*A*) Risk factors for critical care admission. (*B*) Risk factors for mortality.

Male sex was associated with admission to critical care (aOR, 1.81; 95% CI, 1.36–2.40; *P* < .001) (Figure 4*A*), whereas age greater than 70 years showed an inverse association (aOR, 0.47; 95% CI, .27–.83; *P* = .009). No association was seen between coinfection status and critical care admission (aOR, 1.21; 95% CI, .47–3.07; *P* = .69).

Male sex, age >50 years, and increasing number of comorbidities were all associated with increased mortality in univariate and multivariate analysis (Figure 4*B*). However, viral coinfection had no impact on mortality (aOR, 1.17; 95% CI, .31–3.65; *P* = .80).

Ordinal logistic regression analysis demonstrated a significant association between male sex (OR, 1.80; 95% CI, 1.41–2.30; *P* < .001), increasing age (OR, 1.01; 95% CI, 1.00–1.02; *P* = .02), and the presence of 1 comorbidity (OR, 1.43; 95% CI, 1.15–1.79; *P* = .001) with increasing disease severity score (Table 2, Supplementary Table 2, and Supplementary Figure 1), but viral coinfection did not show this association (OR, 1.14; 95% CI, .61–2.14; *P* = .68).

**Table 2.** Ordinal Logistic Regression Analysis of Risk Factors for Increasing COVID-19 Disease Severity Score

Potential risk factor	OR	2.5% CI	97.5% CI	P Value
Coinfection: Yes	1.140	.608	2.135	*P* = .681
Sex: Male	1.801	1.412	2.298	*P* < .001
Age (years)	1.009	1.002	1.017	*P* = .017
Number of comorbidities: 1	1.433	1.149	1.788	*P* = .001
Number of comorbidities: 2+	0.908	.741	1.114	*P* = .356
Immunocompromised: Yes	1.104	.886	1.377	*P* = .376
Timing of sample collection (days since collection of first patient sample)	0.996	.994	.998	*P* < .001

Abbreviations: CI, confidence interval; COVID-19, coronavirus disease 2019; OR, odds ratio.

## DISCUSSION

Viral coinfections were rare (2%) in our multicenter cohort of hospitalized COVID-19 patients recruited during the first 18 months of the pandemic in the UK, with coinfection associated with a variety of respiratory viruses. This is the first study of this size to systematically test hospitalized COVID-19 patients for a broad range of respiratory viruses and as such provides a more accurate picture of viral coinfections among inpatients during this period.

All coinfections were detected in participants admitted before mid-May 2020, with the majority detected in March 2020, and the diversity of viruses detected falling through April and May 2020. This coincides with the introduction of strict public health measures in the UK, including a national lockdown that came into force on March 23, 2020, and correlates with the dramatic decline in the detection of these viruses by national surveillance programs between mid-March and mid-April 2020 [14]. Rhinovirus was the most frequently identified coinfecting virus in our cohort and was also the most ubiquitous virus in the UK in February and early March 2020 [14].

The reported prevalence of viral coinfection in published studies [15–40] vary greatly from 0.5% [17, 32] to 57.3% [22], with our finding of 2% at the lower end of this range. The majority of existing studies identified a prevalence of less than 8%. Studies that reported higher prevalence did not test participants systematically [16, 22, 25, 27, 33, 35, 40], tested solely for influenza [16, 22], used serological methods [22], restricted testing to participants that died [25], or had small study numbers (<500) [22, 25, 27, 33, 35, 40]. With the exception of one study [39], testing for respiratory viruses other than SARS-CoV-2 was carried out at physician discretion or restricted to patients admitted to critical care due to capacity limitations [16]. Consequently, bias towards testing those with severe disease is likely an important confounder. Le Hingrat et al [39] tested all patients hospitalized with influenza-like illness at a “COVID-19 first line hospital” in Paris, France, between January 25 and April 30, 2020, for SARS-CoV-2 and a broad range of other respiratory viruses, and identified coinfection in 6% (49 of 806) of patients, but it was a single-center study over a shorter time period.

Prepandemic viral coinfections were known to be more common in children [41], particularly the under 5-year-olds. A total of 2.2% of our participants were under 18, but this group showed the highest prevalence of viral coinfection (4.5%). This suggests that a similar pattern may be seen in COVID-19 viral coinfection.

However, no significant patient factors, symptoms, or laboratory results were identified as associated with viral coinfection. This illustrates the inability to distinguish between monoinfected and coinfected patients clinically and the need for broad syndromic respiratory viral testing.

Our study showed associations between previously identified risk factors for severe COVID-19 disease, such as male sex and increasing age [9], and admission to critical care or death; however, we did not show such an association with coinfection. This may be due to the low prevalence of coinfected patients identified in our study.

The strength of our study comes from being a large multicenter prospective study with comprehensive clinical metadata encompassing a broad study period. In addition, we used a gold standard diagnostic test, which detects a broad range of endemic respiratory pathogens and is in routine use in the clinical setting, to test for coinfection in a systematic and unbiased manner.

Disentangling the effect of public health measures on virus circulation from potential direct interactions between viruses is difficult. Interactions between cocirculating respiratory viruses can influence patterns of infection at a population level [42]. For example, it has been suggested that the circulation of rhinovirus delayed the spread of pandemic H1N1 influenza A virus in France in 2009 [43], whereas Nickbakhsh et al [42] demonstrated both positive and negative correlations in prevalence between different pairs of viruses even after key alternative drivers of infection such as patient age and seasonal variability were taken into account. Cellular interactions between respiratory viruses during coinfection are likely to result in interlinked patterns of infection at a population level. Several mechanisms of viral interference have been suggested, including the direct blockade of viral entry receptors, viral competition for host cell resources, and the viral induction of immune responses that protect against infection by other viruses [44]. Replication of SARS-CoV-2 in human respiratory epithelial cells has been shown to be inhibited by coinfection with rhinovirus [45], and the interferon response induced by rhinovirus infection has been implicated in protecting against subsequent infection with influenza A [44].

Before COVID-19, viral coinfection had been implicated in exacerbating disease severity [2–6, 46]. However, our study did not demonstrate a significant association between viral coinfection and COVID-19 severity. Although the systematic testing of patients in our study circumvented the selection bias reported in other studies towards increased propensity to test for other respiratory viruses in those with severe disease [38], the small numbers of participants with coinfections will have affected our ability to assess the impact of coinfection on disease severity and identify patient characteristics (risk factors) associated with coinfection.

Animal models of SARS-CoV-2 and influenza A coinfection have demonstrated enhanced disease severity [47, 48], as have 3 large studies of influenza coinfection in COVID-19 patients albeit in cohorts that were not systematically tested for endemic respiratory viruses [15, 16, 38]. We did not detect any coinfection with influenza A in our cohort, thus we could evaluate the impact of influenza coinfection on disease severity in COVID-19.

The unrestricted cocirculation of SARS-CoV-2 with other respiratory viruses is expected to result in a higher incidence of viral coinfections in COVID-19 patients. If coinfections are to become more frequent, then ongoing studies are needed to ascertain the optimal management for these patients. Corticosteroids are recommended in COVID-19 patients requiring supplemental oxygen [49]; a recent Cochrane review showed that corticosteroid treatment is associated with increased risk of mortality and hospital-acquired infection in patients with influenza infection [50]; however, the best approach for coinfected patients is currently unknown.

Our study has several limitations. First, the small numbers of coinfections detected impacted our ability to identify statistical differences between coinfected and monoinfected groups and between those coinfected with different viruses. There was heterogeneity in the interval between symptom onset and when enrollment samples were taken, with a median of 9 days. This could have resulted in the misclassification of participants as having no coinfection if shedding of coinfecting virus had stopped before sampling. Sampling earlier in the course of infection would provide the most accurate measure of coinfection rates and identify true coinfection as opposed to secondary infection. Moreover, the absence of longitudinal samples precluded any analysis of the effect of the order in which infections were acquired. Finally, our cohort of COVID-19 patients were recruited predominantly from the first wave of the pandemic. Only a small proportion of samples (8%, 83 of 1002) were collected between July 2020 and June 2021, which will have restricted our ability to detect coinfections during this period.

## CONCLUSIONS

A low prevalence of viral coinfection was observed in hospitalized COVID-19 patients in the UK during the first 18 months of the pandemic, likely due to strict COVID mitigation measures. However, as these measures are relaxed, and variants continue to evolve, we are seeing a resurgence in endemic respiratory virus circulation. Ongoing respiratory viral surveillance is vital to characterize the interactions between SARS-CoV-2 and endemic respiratory viruses, such as influenza and RSV, and to ascertain the frequency of viral coinfection and its impact on disease severity and healthcare utilization.

## Supplementary Material

ofac531_Supplementary_Data

## Supplementary Data

Supplementary materials are available at *Open Forum Infectious Diseases* online. Consisting of data provided by the authors to benefit the reader, the posted materials are not copyedited and are the sole responsibility of the authors, so questions or comments should be addressed to the corresponding author.

## ISARIC4C Investigators

Co-Lead Investigator (Consortium Lead): J. Kenneth Baillie. Co-Lead Investigator (Imperial College): Peter J. M. Openshaw. *Co-Lead Investigator (Sponsor and Protocol CI): Malcolm G. Semple. Co-Investigator: Beatrice Alex, Petros Andrikopoulos, Benjamin Bach, Wendy S. Barclay, Debby Bogaert, Meera Chand, Kanta Chechi, Graham S. Cooke, Ana da Silva Filipe, Thushan de Silva, Annemarie B. Docherty, Gonçalo dos Santos Correia, Marc-Emmanuel Dumas, Jake Dunning, Tom Fletcher, Christoper A. Green, William Greenhalf, Julian L. Griffin, Rishi K. Gupta, Ewen M. Harrison, Julian A. Hiscox, Antonia Ying Wai Ho, Karl Holden, Peter W. Horby, Samreen Ijaz, Saye Khoo, Paul Klenerman, Andrew Law, Matthew R. Lewis, Sonia Liggi, Wei Shen Lim, Lynn Maslen, Alexander J. Mentzer, Laura Merson, Alison M. Meynert, Shona C. Moore, Mahdad Noursadeghi, Michael Olanipekun, Anthonia Osagie, Massimo Palmarini, Carlo Palmieri, William A. Paxton, Georgios Pollakis, Nicholas Price, Andrew Rambaut, David L. Robertson, Clark D. Russell, Vanessa Sancho-Shimizu, Caroline J. Sands, Janet T. Scott, Louise Sigfrid, Tom Solomon, Shiranee Sriskandan, David Stuart, Charlotte Summers, Olivia V. Swann, Zoltan Takats, Panteleimon Takis, Richard S. Tedder, A. A. Roger Thompson, Emma C. Thomson, Ryan S Thwaites, Lance CW Turtle, Maria Zambon. Data Analyst: Thomas M. Drake, Cameron J. Fairfield, Stephen R. Knight, Kenneth A. Mclean, Derek Murphy, Lisa Norman, Riinu Pius, Catherine A. Shaw. Data and Information System Manager: Marie Connor, Jo Dalton, Carrol Gamble, Michelle Girvan, Sophie Halpin, Janet Harrison, Clare Jackson, James Lee, Laura Marsh, Daniel Plotkin, Stephanie Roberts, Egle Saviciute. Data Integration and Presentation: Sara Clohisey, Ross Hendry, Susan Knight, Eva Lahnsteiner, Andrew Law, Gary Leeming, Lucy Norris, James Scott-Brown, Sarah Tait, Murray Wham. ISARIC Clinical Coordinator: Gail Carson. Edinburgh Laboratory Staff and Volunteers: Richard Clark, Audrey Coutts, Lorna Donnelly, Angie Fawkes, Tammy Gilchrist, Katarzyna Hafezi, Louise MacGillivray, Alan Maclean, Sarah McCafferty, Kirstie Morrice, Lee Murphy, Nicola Wrobel. Local Principal Investigators: Kayode Adeniji, Daniel Agranoff, Ken Agwuh, Dhiraj Ail, Erin L. Aldera, Ana Alegria, Sam Allen, Brian Angus, Abdul Ashish, Dougal Atkinson, Shahedal Bari, Gavin Barlow, Stella Barnass, Nicholas Barrett, Christopher Bassford, Sneha Basude, David Baxter, Michael Beadsworth, Jolanta Bernatoniene, John Berridge, Colin Berry, Nicola Best, Pieter Bothma, Robin Brittain-Long, Naomi Bulteel, Tom Burden, Andrew Burtenshaw, Vikki Caruth, David Chadwick, David Chadwick, Duncan Chambler, Nigel Chee, Jenny Child, Srikanth Chukkambotla, Tom Clark, Paul Collini, Catherine Cosgrove, Jason Cupitt, Maria-Teresa Cutino-Moguel, Paul Dark, Chris Dawson, Samir Dervisevic, Phil Donnison, Sam Douthwaite, Andrew Drummond, Ingrid DuRand, Ahilanadan Dushianthan, Tristan Dyer, Cariad Evans, Chi Eziefula, Chrisopher Fegan, Adam Finn, Duncan Fullerton, Sanjeev Garg, Sanjeev Garg, Atul Garg, Effrossyni Gkrania-Klotsas, Jo Godden, Arthur Goldsmith, Clive Graham, Tassos Grammatikopoulos, Elaine Hardy, Stuart Hartshorn, Daniel Harvey, Peter Havalda, Daniel B. Hawcutt, Maria Hobrok, Luke Hodgson, Anil Hormis, Joanne Howard, Michael Jacobs, Susan Jain, Paul Jennings, Agilan Kaliappan, Vidya Kasipandian, Stephen Kegg, Michael Kelsey, Jason Kendall, Caroline Kerrison, Ian Kerslake, Oliver Koch, Gouri Koduri, George Koshy, Shondipon Laha, Steven Laird, Susan Larkin, Tamas Leiner, Patrick Lillie, James Limb, Vanessa Linnett, Jeff Little, Mark Lyttle, Michael MacMahon, Emily MacNaughton, Ravish Mankregod, Huw Masson, Elijah Matovu, Katherine McCullough, Ruth McEwen, Manjula Meda, Gary Mills, Jane Minton, Mariyam Mirfenderesky, Kavya Mohandas, Quen Mok, James Moon, Elinoor Moore, Patrick Morgan, Craig Morris, Katherine Mortimore, Samuel Moses, Mbiye Mpenge, Rohinton Mulla, Michael Murphy, Thapas Nagarajan, Megan Nagel, Mark Nelson, Lillian Norris, Matthew K. O'Shea, Marlies Ostermann, Igor Otahal, Mark Pais, Carlo Palmieri, Selva Panchatsharam, Danai Papakonstantinou, Padmasayee Papineni, Hassan Paraiso, Brij Patel, Natalie Pattison, Justin Pepperell, Mark Peters, Mandeep Phull, Stefania Pintus, Tim Planche, Frank Post, David Price, Rachel Prout, Nikolas Rae, Henrik Reschreiter, Tim Reynolds, Neil Richardson, Mark Roberts, Devender Roberts, Alistair Rose, Guy Rousseau, Bobby Ruge, Brendan Ryan, Taranprit Saluja, Sarah Cole, Matthias L. Schmid, Aarti Shah, Manu Shankar-Hari, Prad Shanmuga, Anil Sharma, Anna Shawcross, Jagtur Singh Pooni, Jeremy Sizer, Richard Smith, Catherine Snelson, Nick Spittle, Nikki Staines, Tom Stambach, Richard Stewart, Pradeep Subudhi, Tamas Szakmany, Kate Tatham, Jo Thomas, Chris Thompson, Robert Thompson, Ascanio Tridente, Darell Tupper-Carey, Mary Twagira, Nick Vallotton, Rama Vancheeswaran, Lisa Vincent-Smith, Shico Visuvanathan, Alan Vuylsteke, Sam Waddy, Rachel Wake, Andrew Walden, Ingeborg Welters, Tony Whitehouse, Paul Whittaker, Ashley Whittington, Meme Wijesinghe, Martin Williams, Lawrence Wilson, Stephen Winchester, Martin Wiselka, Adam Wolverson, Daniel G. Wootton, Andrew Workman, Bryan Yates, Peter Young. Material Management: Sarah E. McDonald, Victoria Shaw. Outbreak Laboratory Staff and Volunteers: Katie A. Ahmed, Jane A. Armstrong, Milton Ashworth, Innocent G. Asiimwe, Siddharth Bakshi, Samantha L. Barlow, Laura Booth, Benjamin Brennan, Katie Bullock, Nicola Carlucci, Emily Cass, Benjamin W. A. Catterall, Jordan J. Clark, Emily A. Clarke, Sarah Cole, Louise Cooper, Helen Cox, Christopher Davis, Oslem Dincarslan, Alejandra Doce Carracedo, Chris Dunn, Philip Dyer, Angela Elliott, Anthony Evans, Lorna Finch, Lewis W. S. Fisher, Lisa Flaherty, Terry Foster, Isabel Garcia-Dorival, Philip Gunning, Catherine Hartley, Anthony Holmes, Rebecca L. Jensen, Christopher B. Jones, Trevor R. Jones, Shadia Khandaker, Katharine King, Robyn T. Kiy, Chrysa Koukorava, Annette Lake, Suzannah Lant, Diane Latawiec, Lara Lavelle-Langham, Daniella Lefteri, Lauren Lett, Lucia A. Livoti, Maria Mancini, Hannah Massey, Nicole Maziere, Sarah McDonald, Laurence McEvoy, John McLauchlan, Soeren Metelmann, Nahida S. Miah, Joanna Middleton, Joyce Mitchell, Shona C. Moore, Ellen G. Murphy, Rebekah Penrice-Randal, Jack Pilgrim, Tessa Prince, Will Reynolds, P. Matthew Ridley, Debby Sales, Victoria E. Shaw, Rebecca K. Shears, Benjamin Small, Krishanthi S. Subramaniam, Agnieska Szemiel, Aislynn Taggart, Jolanta Tanianis-Hughes, Jordan Thomas, Erwan Trochu, Libby van Tonder, Eve Wilcock, J. Eunice Zhang. Patient Engagement: Seán Keating. Project Administrator: Cara Donegan, Rebecca G. Spencer. Project Manager: Chloe Donohue, Fiona Griffiths, Hayley Hardwick, Wilna Oosthuyzen.
