# Supplementary material for: Viral Coinfections in Hospitalized Coronavirus Disease 2019 Patients Recruited to the International Severe Acute Respiratory and Emerging Infections Consortium WHO Clinical Characterisation Protocol UK Study
Source: Open Forum Infect Dis. 2022 Oct 10;9(11):ofac531. doi: 10.1093/ofid/ofac531 (PMC9619746; doi:10.1093/ofid/ofac531)
Supplement: ofac531_Supplementary_Data [file ofac531_supplementary_data.zip › Vink_viral_coinfection_in_covid19_Supplementary_Figure 1.docx]

### Supplementary Figure 1: a) QQ plot for Ordinal Logistic Regression Model; b) ecdf(x) plot for ordinal logistic regression model.
