# Supplementary material for: Viral Coinfections in Hospitalized Coronavirus Disease 2019 Patients Recruited to the International Severe Acute Respiratory and Emerging Infections Consortium WHO Clinical Characterisation Protocol UK Study
Source: Open Forum Infect Dis. 2022 Oct 10;9(11):ofac531. doi: 10.1093/ofid/ofac531 (PMC9619746; doi:10.1093/ofid/ofac531)
Supplement: ofac531_Supplementary_Data [file ofac531_supplementary_data.zip › Vink_viral_coinfection_in_covid19_Supplementary_Table_1.docx]

### Supplementary Table 1: Symptoms at admission, by co-infection status

|  | **n (% of total)** | **All patients** |  | **Co-infected** | **Not Co-infected** | **p-value** |
| --- | --- | --- | --- | --- | --- | --- |
| Symptom duration at admission (days) | 971 (96.9) | 7.0  (3.0 to 10.0) |  | 7.0  (3.0 to 8.0) | 7.0  (3.0 to 10.0) | 0.984 |
| Cough | 972 (97.0) | 770 (79.2) |  | 15 (75.0) | 755 (79.3) | 0.585 |
| Fever | 966 (96.4) | 734 (76.0) |  | 14 (70.0) | 720 (76.1) | 0.596 |
| Shortness of Breath | 954 (95.2) | 706 (74.0) |  | 13 (68.4) | 693 (74.1) | 0.599 |
| Fatigue | 849 (84.7) | 430 (50.6) |  | 7 (43.8) | 423 (50.8) | 0.622 |
| Myalgia | 823 (82.1) | 252 (30.6) |  | 3 (20.0) | 249 (30.8) | 0.572 |
| Sputum Production | 870 (86.8) | 244 (28.0) |  | 4 (22.2) | 240 (28.2) | 0.792 |
| Diarrhoea | 884 (88.2) | 222 (25.1) |  | 5 (29.4) | 217 (25.0) | 0.777 |
| Vomiting | 873 (87.1) | 200 (22.9) |  | 2 (11.8) | 198 (23.1) | 0.386 |
| Headache | 820 (81.8) | 159 (19.4) |  | 1 (6.2) | 158 (19.7) | 0.333 |
| Confusion | 893 (89.1) | 132 (14.8) |  | 4 (21.1) | 128 (14.6) | 0.508 |
| Sore Throat | 807 (80.5) | 103 (12.8) |  | 0 (0.0) | 103 (13.0) | 0.239 |
| Abdominal Pain | 860 (85.8) | 83 (9.7) |  | 1 (5.6) | 82 (9.7) | 1.000 |
| Wheeze | 832 (83.0) | 67 (8.1) |  | 3 (18.8) | 64 (7.8) | 0.131 |
| Runny Nose | 798 (79.6) | 47 (5.9) |  | 1 (6.7) | 46 (5.9) | 0.601 |
| Rash | 844 (84.2) | 16 (1.9) |  | 0 (0.0) | 16 (1.9) | 1.000 |
| Conjunctivitis | 830 (82.8) | 2 (0.2) |  | 0 (0.0) | 2 (0.2) | 1.000 |

Data are n(%) or median (IQR)
