# Supplementary material for: Viral Coinfections in Hospitalized Coronavirus Disease 2019 Patients Recruited to the International Severe Acute Respiratory and Emerging Infections Consortium WHO Clinical Characterisation Protocol UK Study
Source: Open Forum Infect Dis. 2022 Oct 10;9(11):ofac531. doi: 10.1093/ofid/ofac531 (PMC9619746; doi:10.1093/ofid/ofac531)
Supplement: ofac531_Supplementary_Data [file ofac531_supplementary_data.zip › Vink_viral_coinfection_in_covid19_Supplementary_Table_2.docx]

### Supplementary Table 2: Ordinal Logistic Regression Model Outputs

|  | **Value** | **Std. Error** | **T value** | **P value** | **2.50%** | **97.50%** |
| --- | --- | --- | --- | --- | --- | --- |
| **Coefficients** |  |  |  |  |  |  |
| Co-infection: Yes | 0.131 | 0.319 | 0.411 | 0.681 | -0.498 | 0.758 |
| Sex: Male | 0.588 | 0.124 | 4.740 | <0.001 | 0.346 | 0.832 |
| Age (years) | 0.009 | 0.004 | 2.388 | 0.017 | 0.002 | 0.017 |
| Number of Comorbidities: 1 | 0.360 | 0.113 | 3.190 | 0.001 | 0.139 | 0.581 |
| Number of Comorbidities: 2+ | -0.096 | 0.104 | -0.922 | 0.356 | -0.300 | 0.108 |
| Immunocompromised: Yes | 0.100 | 0.112 | 0.886 | 0.376 | -0.121 | 0.320 |
|  | -0.004 | 0.001 | -3.411 | <0.001 | -0.006 | -0.002 |
| **Intercepts** |  |  |  |  |  |  |
| 1\|2 | -0.971 | 0.346 | -2.803 | 0.005 |  |  |
| 2\|3 | 0.381 | 0.344 | 1.106 | 0.269 |  |  |
| 3\|4 | 1.173 | 0.345 | 3.404 | <0.001 |  |  |
| 4\|5 | 2.045 | 0.349 | 5.859 | <0.001 |  |  |
|  |  |  |  |  |  |  |
| Residual Deviance | 2814.084 |  |  |  |  |  |
| AIC | 2836.084 |  |  |  |  |  |
